# Missing Rings, Synchronous Growth, and Ecological Disturbance in a 36-Year Pitch Pine (*Pinus rigida*) Provenance Study

**DOI:** 10.1371/journal.pone.0154730

**Published:** 2016-05-16

**Authors:** Caroline Leland, John Hom, Nicholas Skowronski, F. Thomas Ledig, Paul J. Krusic, Edward R. Cook, Dario Martin-Benito, Javier Martin-Fernandez, Neil Pederson

**Affiliations:** 1 Lamont-Doherty Earth Observatory of Columbia University, Palisades, New York, United States of America; 2 United States Department of Agriculture Forest Service, Northern Research Station, Newtown Square, Pennsylvania, United States of America; 3 United States Department of Agriculture Forest Service, Northern Research Station, Morgantown, West Virginia, United States of America; 4 Department of Plant Sciences, University of California, Davis, California, United States of America; 5 Stockholm University, Department of Physical Geography and Quaternary Geology, Stockholm, Sweden; 6 Navarino Environmental Observatory, Messinia, Greece; 7 Institute of Terrestrial Ecosystems, ETH Zürich, Zürich, Switzerland; 8 Harvard University, Harvard Forest, Petersham, Massachusetts, United States of America; Technical University in Zvolen, SLOVAKIA

## Abstract

Provenance studies are an increasingly important analog for understanding how trees adapted to particular climatic conditions might respond to climate change. Dendrochronological analysis can illuminate differences among trees from different seed sources in terms of absolute annual growth and sensitivity to external growth factors. We analyzed annual radial growth of 567 36-year-old pitch pine (*Pinus rigida* Mill.) trees from 27 seed sources to evaluate their performance in a New Jersey Pine Barrens provenance experiment. Unexpectedly, missing rings were prevalent in most trees, and some years—1992, 1999, and 2006—had a particularly high frequency of missing rings across the plantation. Trees from local seed sources (<55 km away from the plantation) had a significantly smaller percentage of missing rings from 1980–2009 (mean: 5.0%), relative to northernmost and southernmost sources (mean: 9.3% and 7.9%, respectively). Some years with a high frequency of missing rings coincide with outbreaks of defoliating insects or dry growing season conditions. The propensity for missing rings synchronized annual variations in growth across all trees and might have complicated the detection of potential differences in interannual variability among seed sources. Average ring width was significantly larger in seed sources from both the southernmost and warmest origins compared to the northernmost and coldest seed sources in most years. Local seed sources had the highest average radial growth. Adaptation to local environmental conditions and disturbances might have influenced the higher growth rate found in local seed sources. These findings underscore the need to understand the integrative impact of multiple environmental drivers, such as disturbance agents and climate change, on tree growth, forest dynamics, and the carbon cycle.

## Introduction

Provenance trial studies assess the relative success of trees from different seed sources planted in a common plantation. As global climate change is expected to have local impacts on forest dynamics and productivity, provenance studies are useful for understanding species-specific responses to climatic change. Transferring seedlings from a region in which they are adapted to a new location simulates an abrupt change in climate. Consequently, these studies can indicate how a particular species might fare under altered environmental conditions. Seed source performance within the plantation is often evaluated with metrics such as tree height, bole volume, phenology, serotiny, or survival and fecundity (e.g. [1,2]). Seed source performance can also be assessed through comparisons with trees from local seed sources, which are expected to be best adapted to the climatic and environmental conditions of the test site [3]. However, some populations can benefit from being transferred to a region with different climatic conditions (e.g.[4,5]), suggesting that local seed sources might not always perform best under future climate change scenarios. The information gleaned from provenance trial studies can guide forest managers when selecting seed sources for local reforestation [6,7].

Only a few provenance experiments have evaluated seed source performance using tree-ring methods (e.g. [8–10]). Tree-ring analysis can complement common forest mensuration metrics by providing information on radial growth patterns, trends, and response to climate over time. These studies illustrate how tree provenance (and presumed genetic differences) could influence a tree’s growth response to climate in the plantation setting. Using this approach, McLane et al. [9] found differences in the growth-climate sensitivity among seed provenances of *Pinus contorta* (Dougl. var. *latafolia* Engelm.) across British Columbia and Yukon Territory, Canada where trees originating from warm sites were more sensitive to climate in colder sites and vice versa. These results suggest that genetics can play an important role in forest productivity under a warming climate. Similarly, Savva et al. [10] found that some distant and southern seed sources from southern Canada and the northern United States had higher mean growth relative to local populations in an Ontario, Canada provenance study. In contrast, Cook et al. [8] found relatively small to insignificant differences among seed sources of loblolly pine (*Pinus taeda* L.) growing within individual plantations across the southern United States.

**Fig 1:**
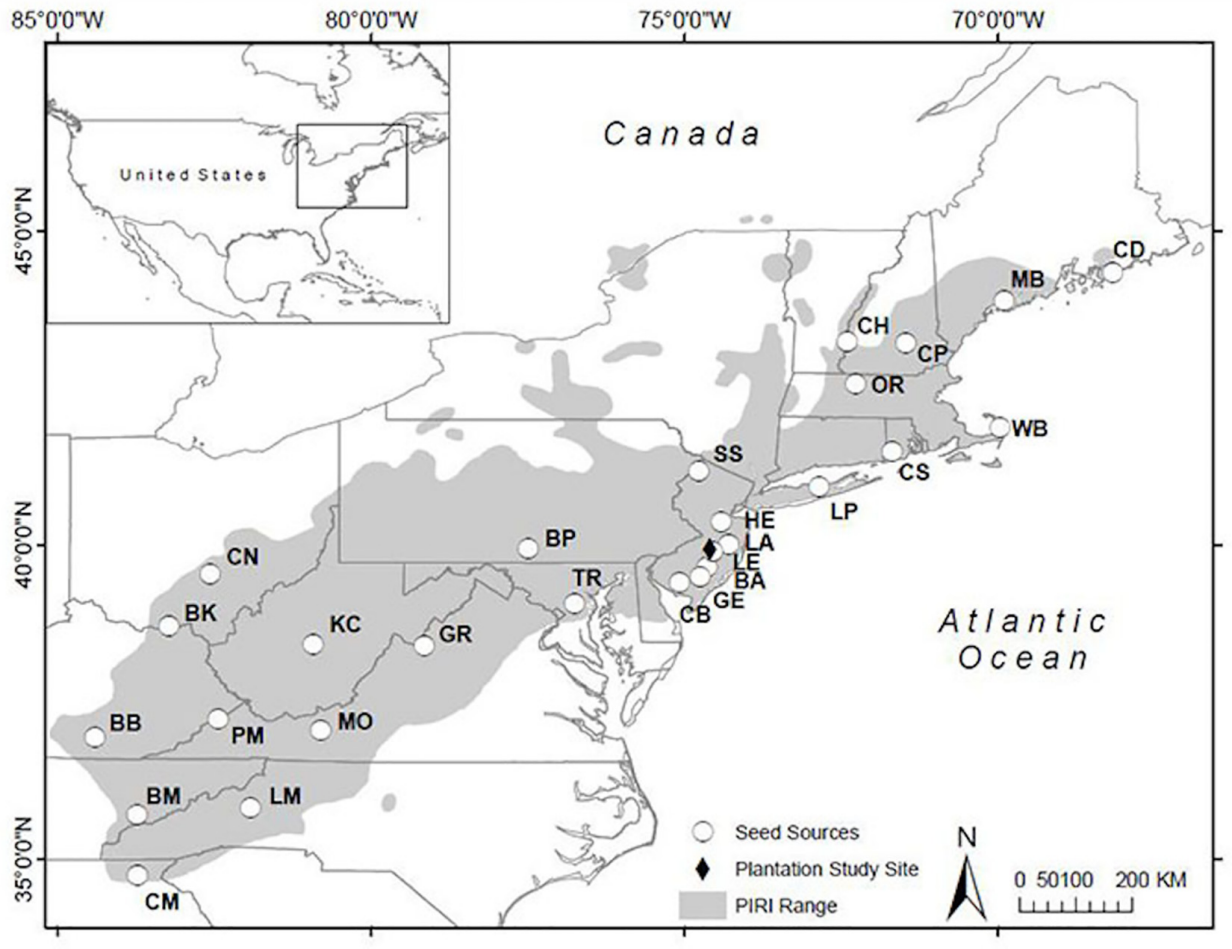
Map of 27 *Pinus rigida* seed sources from the provenance study. The diamond indicates the location of the Brendan T. Byrne plantation site in New Jersey. Light gray represents the *P*. *rigida* range [48].

Here, we evaluated trees from a pitch pine (*Pinus rigida* Mill.) provenance study established in 1974 in the New Jersey Pinelands National Reserve. We used tree-ring analysis to compare annual growth of trees propagated from 27 distinct seed sources spanning an area covering nearly 10 degrees of latitude and 16 degrees of longitude in the eastern United States (Fig 1; [11,12]). Our objective was to determine whether absolute radial growth, or annual growth sensitivity to local climate, differed among seed sources.

## Materials and Methods

In May 2010, 953 pitch pine trees were cored in a provenance plantation in the Brendan T. Byrne State Forest (formerly known as the Lebanon State Forest) in Ocean County, New Jersey, USA. Christian M. Bethmann of the New Jersey Division of Parks and Forestry, and superintendent of the Brendan T. Byrne State Forest, granted sampling permission. Trees in the plantation site were growing on sandy and well-drained soil. The provenance plantation was designed as a compact family block, with seed sources randomized in each block, and families randomized within each seed source [11]. The pitch pine trees for this study originated from 27 seed sources spanning 34.74–44.33° N and 68.18–84.45° W (Fig 1, Table 1). Six of the largest trees were selected for coring in each seed source plot. Using a gas-powered drill adapted for an increment borer, each tree was cored bark-to-bark (i.e. one core through the entire stem) such that two diametrically opposed radii were collected at the same time. Cores were taken <0.2 m above the root collar of each tree. Core samples were prepared using standard dendrochronological techniques [13] and scanned at 1200 dots per inch for image analysis.

**Table 1:** List of seed sources and relevant statistics.

Site	State	Distance from Plantation (km)	# Series	Series Intercorr.^a^	Total Missing Rings (%)^b^	1992 Missing Ring (%)^c^	EPS^d^
Cadillac Cliffs (CD)	ME	721	42	0.644	6.35	42.86	0.988
Mare Brook (MB)	ME	587	42	0.643	9.21	71.42	0.989
Charlestown (CH)	NH	413	42	0.679	9.68	95.23	0.989
Concord Plains (CP)	NH	449	41	0.676	10.79	95.24	0.986
Orange (OR)	MA	352	40	0.688	10.48	100	0.987
Wellfleet Bay (WB)	MA	445	41	0.718	5.71	61.90	0.990
Carolina State Forest (CS)	RI	300	42	0.710	8.10	61.90	0.988
Stokes State Forest (SS)	NY	142	42	0.705	9.05	95.24	0.989
Long Pond Road (LP)	NY	184	41	0.726	6.03	76.19	0.990
Lebanon Lakes (LE)	NJ	9	41	0.757	6.03	71.43	0.989
Helmetta (HE)	NJ	53	41	0.703	4.44	76.19	0.991
Lakehurst (LA)	NJ	27	42	0.733	5.08	66.67	0.987
Big Pine Flat Ridge (BP)	PA	247	40	0.680	6.14	78.95	0.985
*VOLUNTEER*	—	-	42	0.742	4.13	47.62	0.990
Batsto (BA)	NJ	30	40	0.749	3.63	72.73	0.988
Cantwell Cliffs (CN)	OH	684	41	0.635	8.73	90.48	0.988
Great Egg Harbor River (GE)	NJ	48	39	0.718	5.87	85.71	0.991
Cumberland (CB)	NJ	71	41	0.741	7.14	95.24	0.990
Trainfire Road (TR)	MD	209	42	0.642	8.89	76.19	0.991
Bald Knob Run (BK)	OH	754	40	0.668	6.35	76.19	0.989
Kyle Cemetery (KC)	WV	571	41	0.695	6.19	71.43	0.990
Grooms Ridge (GR)	VA	429	42	0.639	8.89	95.24	0.992
Pine Mountain (PM)	KY	744	41	0.640	6.83	80.95	0.985
Mount Olivet (MO)	VA	628	41	0.676	11.75	94.74	0.985
Big Bend (BB)	KY	916	41	0.691	7.04	83.33	0.989
Linville Mountain (LM)	NC	788	40	0.626	8.41	71.43	0.988
Bates Mountain (BM)	TN	928	41	0.626	5.40	61.90	0.985
Crumbly Mountain (CM)	GA	991	42	0.615	7.30	66.67	0.991

_a:_ Average series intercorrelation based on Spearman’s rank correlations.

_b:_ Percentage of missing rings in all trees calculated over the 1980–2009 period. If a ring was present along one radius (series) of a tree, but absent in the other, it was counted as present overall.

_c:_ Percentage of trees missing the 1992 growth ring. If the 1992 ring was present along one radius (series) of a tree, but absent in the other, it was counted as present overall.

_d:_ The Expressed Population Signal (EPS) calculated over the 1980–2009 period.

Scanned cores were cross-dated and measured to 0.001 mm precision using WinDendro software version 2009b [14]. Missing (locally absent), micro and false rings were a common feature in the plantation trees. Due to the limitations of image analysis technology for identifying these features, cross dating in problem areas was verified with a stereo microscope [15]. Common latewood variation and false rings patterns among trees were especially important for visual cross-dating. Ring-width measurements were statistically tested for cross-dating precision using the program COFECHA [16].

Subsequent analyses were limited to 21 randomly selected trees from each seed source because Bates Mountain (BM) had the least number of sampled trees (n = 21). In some cases, due to occasional damage, only one series was retained per tree. As a result, the number of radii analyzed per seed source varied from 39–42 series (Table 1), leading to a total of 567 trees and 1,109 series used for analysis.

The high frequency of missing rings prompted a collection of samples from 21 trees that naturally regenerated adjacent to the plantation (the Volunteer population). Volunteer trees were destructively sampled using a chainsaw and disc samples were collected at one-meter intervals along the stem of each tree. The bottom cross-section of the Volunteer trees had an average pith date of 1976 (min: 1975; max: 1978), thus, it is most probable that the Volunteer trees were derived from extant, local, and mature pitch pine trees. The basal (~0.2 meter height), middle (~5 meters height) and near-top (~8 meters height) stem discs from each Volunteer tree were analyzed to quantify the frequency of missing rings as a function of tree height. Each disc was divided into eighths and rings were classified as fully present, pinching (partially present), or entirely missing in each eighth section. Ring-width data from the Volunteer basal samples were compared to each seed source because the basal height was nearly equivalent to coring height for the plantation trees.

Differences in the percentage of missing rings and average radial growth among seed sources were examined in relation to seed source May-September average temperature and precipitation (derived from PRISM monthly normals from 1981–2010 [17]), latitude, and distance from the plantation site. Seed sources were grouped into the five northernmost (CD, MB, CH, CP, OR), southernmost (CM, MO, BB, LM, BM), coldest (CD, MB, CH, CP, OR), warmest (BA, CB, BM, TR, BB), local (LE, HE, LA, BA, GE; all<55 km away) and most distant (CM, BM, BB, LM, BK) seed sources. Some groups are similar in their seed source composition (e.g. most distant and southernmost) or identical (northernmost and coldest), therefore, these groups were not directly compared. A Kruskal-Wallis H test and Wilcoxon rank-sum tests were used to test whether there were statistically significant differences in the percentage of missing rings between trees from different seed source groups. When using the Kruskal-Wallis H test for comparing more than two groups, pairwise comparisons were conducted using Wilcoxon rank-sum tests with a Bonferroni p-value correction. Because sample replication began to decline prior to 1980 (i.e. not all cores captured the earliest growth rings), the percentage of missing rings was calculated relative to the 1980–2009 period. A year was considered missing only if the growth ring was absent on both radii from each tree. Next, we used bootstrap resampling, with 1000 replicates, to estimate 95% confidence intervals for annual ring-width measurements averaged across all trees from a given seed source group for the 1980–2009 period. To compare a pair of seed source groups, we determined annual mean ring-width values to be significantly different if there was ≤5% overlap between the two sets of 1000 randomly ordered bootstrap replicate values.

To compare interannual growth variability and climate response among seed sources, each series of raw measurements from a provenance was standardized to remove potential non-climatic growth trends, such as those related to the allometric growth trend, using the program ARSTAN [18]. Prior to standardization, radial measurements were power transformed to minimize heteroscedastic variance often found in ring-width measurements [19]. After transformation, a cubic smoothing spline with a 50% frequency response cut-off equal to 2/3 the series length was fit to the power-transformed series. The residuals from the spline fit were used to produce a biweight robust mean chronology for each seed source. Only the mean chronologies from 1980–2009 were used in further analyses to avoid reduced tree replication and potential transplant shock.

We performed a principal component analysis (PCA) on the chronologies to identify unique modes of variation within all of the seed sources, and a Rule-N test was used for calculating the number of significant components. To evaluate the response of tree growth to climate, we calculated Pearson correlations between the average chronology index of all seed sources and total monthly precipitation and average maximum temperature from 1980–2009. The years 1992–1994 were not included in these analyses because of strong evidence indicating a severe insect defoliation and slow recovery within the stand. The meteorological data were taken from two stations within 20 km of the plantation site: the nearest for monthly maximum average temperatures was the McGuire Air Force Base, and the nearest for precipitation data was the Indian Mills meteorological station.

## Results

### Tree-ring analysis and missing rings

**Fig 2:**
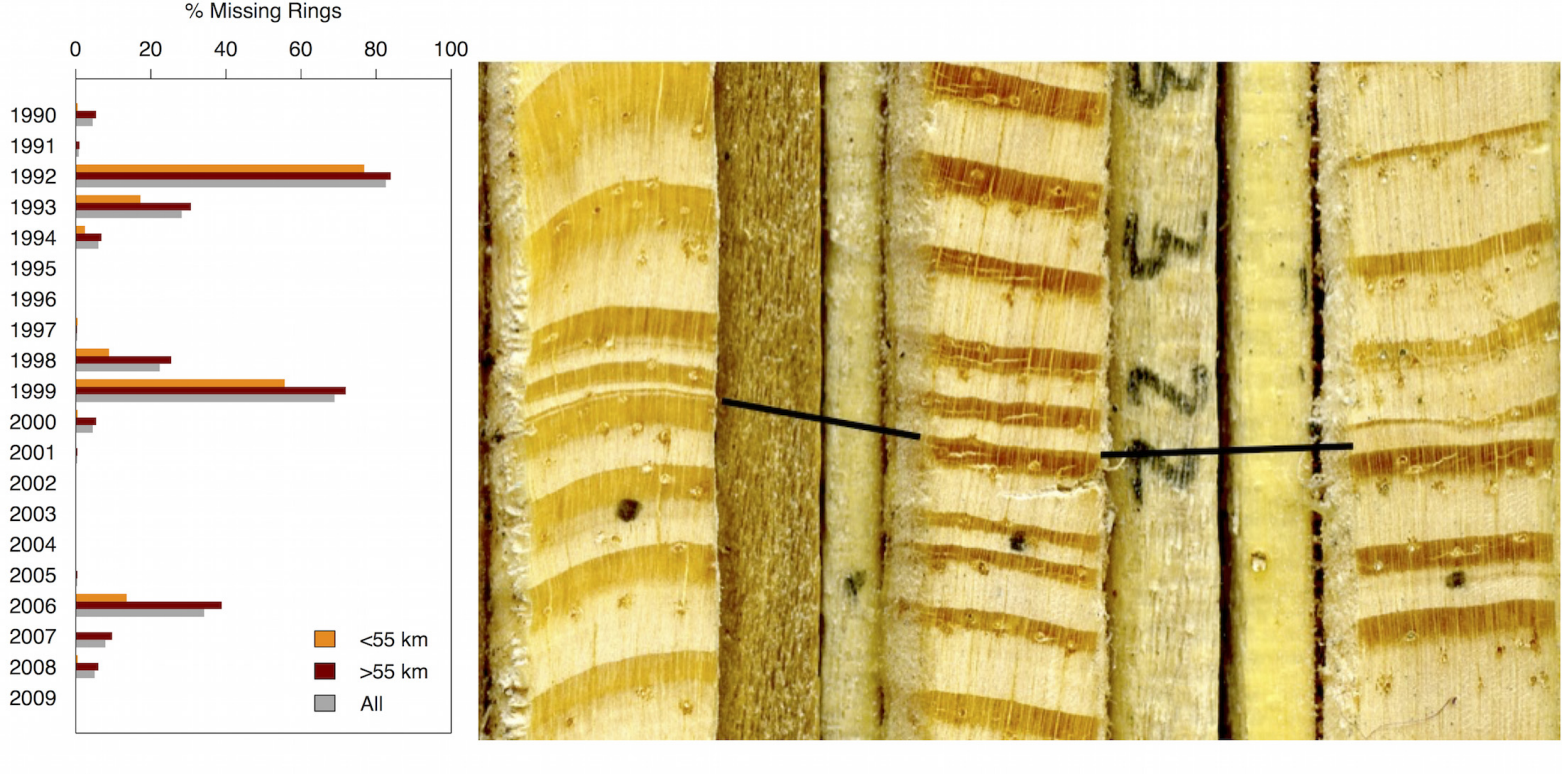
Missing rings. Left: Annual percentage of total missing rings across all series (including all seed sources; gray), local seed sources (< 55 km away from the plantation site; orange), and distant seed sources (>55 km from the plantation; red). Right: Scanned image of pitch pine cores with lines pointing to the year 1992, which is apparent in the far-left tree core as a micro ring, almost imperceptible in the middle core, and clearly evident in the far right core.

Missing growth rings were common in all seed sources and initially complicated the cross-dating process. Years with a high frequency of missing rings included 1990, 1992–1994, 1998–2000, and 2006–2008 (Fig 2). There were no missing rings prior to 1990 and local seed sources (<55 km from the plantation) had a lower percentage of missing rings relative to all other seed sources in all years. The year 1992 had the highest frequency of missing rings, with 42.86–100% of trees missing a ring within individual seed sources (Table 1). In fact, approximately 180 trees (~360 or 19% of all series) were examined before it was determined that a growth ring for this year was missing in most samples. When present, 1992 was particularly narrow (Fig 2). The year 1999 had the second highest frequency of missing rings with 69% of series missing this ring across all seed sources. Although several individual trees had no missing rings, there were no seed source collections without missing rings. From 1980–2009, Batsto (BA) had the lowest percentage of missing rings across all trees (3.63%), and Mount Olivet (MO) had the highest (11.75%). Growing adjacent to the plantation, the Volunteer trees had the second lowest percentage of missing rings in comparison to all seed sources (4.13% from 1980–2009).

**Fig 3:**
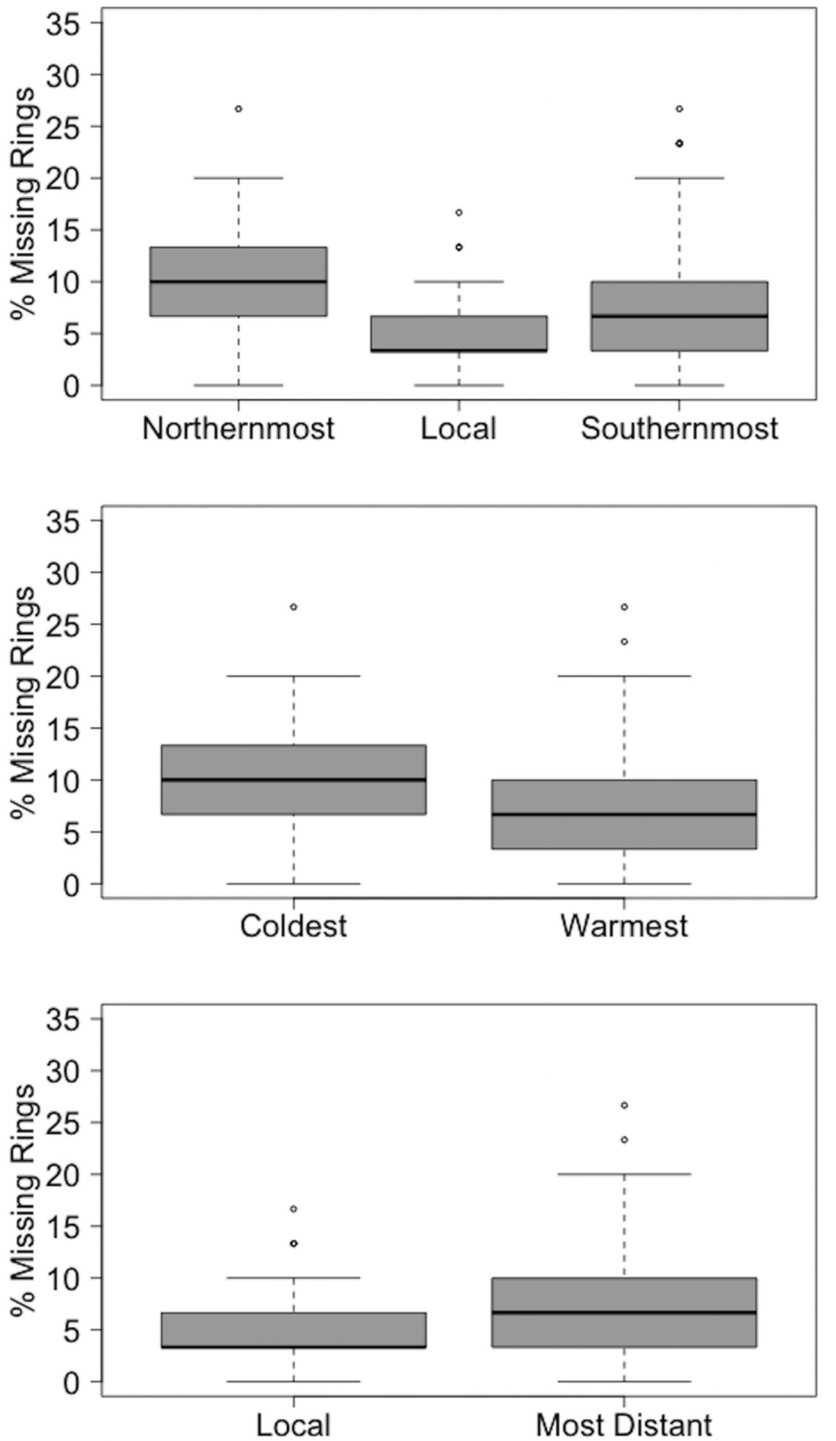
Percentage of missing rings for different seed source groups. The percentage of missing rings for all trees from the five northernmost, southernmost, local, coldest, warmest, and most distant seed sources. The percentage of missing rings for each tree was determined over the period 1980–2009.

In comparing missing rings between seed sources groups (Fig 3), the five northernmost, southernmost, and local (central) seed sources had a significantly different percentage of missing rings (Kruskal-Wallis H-test; *p* < 0.001). Local seed sources have a significantly lower percentage of missing rings relative to northernmost and southernmost seed sources (pairwise Wilcoxon test: *p* < 0.001 and *p* = 0.007, respectively). Northern and southern seed sources did not have a significantly different percentage of missing rings (pairwise Wilcoxon Test: *p* = 0.09). However, the coldest seed sources had a higher percentage of missing rings than the warmest seed sources (Wilcoxon Test: *p* < 0.001). Local seed sources had a lower percentage of missing rings than the most distant seed sources, though this result was marginally statistically significant (Wilcoxon Test: *p* = 0.049).

### Stem analysis

**Fig 4:**
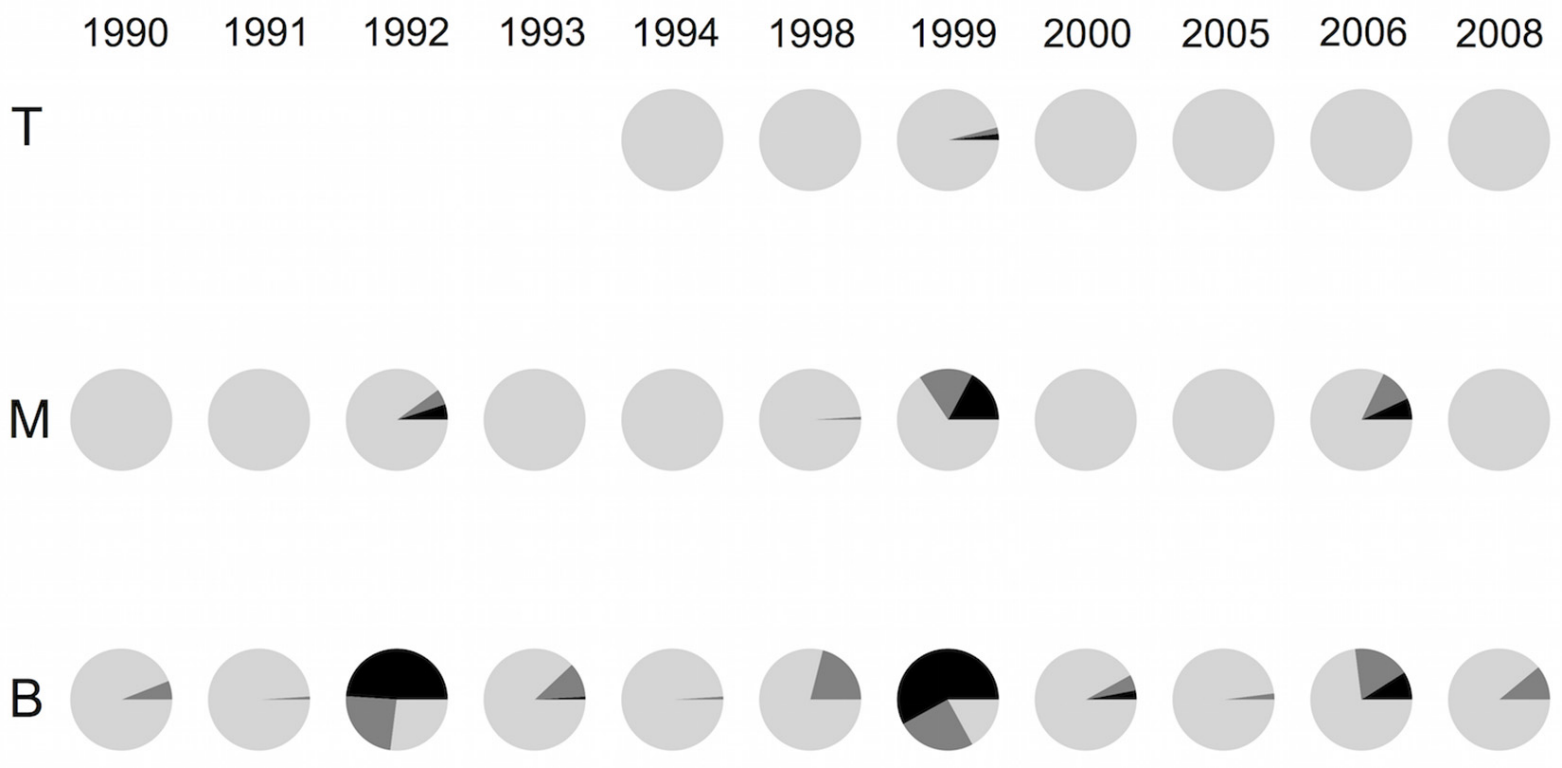
Volunteer stem analysis. The percentage of total eighth sections showing absent (black), pinching (medium gray), or fully present (light gray) rings for the discs along different heights of the stem of Volunteer trees. The basal (B), middle (M), and top (T) sections are shown. Fewer than six trees extended before 1994 for the top disc sample, therefore we truncated the analysis at that year for the top stem sample only. All of the basal and middle sections extended prior to 1990.

Analysis of samples at one-meter intervals along the stems of Volunteer trees illustrate that the propensity for missing rings changes as a function of height along the tree bole (Fig 4). An analysis of fully present, pinching, and absent rings within each eighth area section of each disk sample from the base, middle, and top of the Volunteer trees reveals that 1992, 1999, and 2006 had the highest amount of pinching and fully missing rings in the basal and middle sections of the stem; many missing or pinching rings on the lower part of the stem became full rings as stem height increased. Only 17% of the analyzed sections had a fully present 1999 ring in the basal samples, but this increased to 95% in the top samples. Similarly, 73% of the sections had a full 2006 ring in the basal section, which increased to 100% in the top samples.

### Growth analysis

In viewing all seed sources along latitudinal and climatological gradients, there is considerable variability in average ring width among trees of individual seed sources (S1 Fig). However, at the overall seed source scale, average ring width tends to increase with decreasing latitude and increasing seed source temperature (S1 Fig). These trends are interrupted by high growth rates associated with local and Volunteer seed sources (local seed source latitude range: ~39.5–40.4°N, and temperature range: ~20.7–21.2°C). There is no clear relationship between mean tree-ring width of each seed source and provenance precipitation. Local seed sources have larger average ring widths, but beyond this local region, there is no apparent relationship between distance from plantation and average radial growth (S1 Fig).

**Fig 5:**
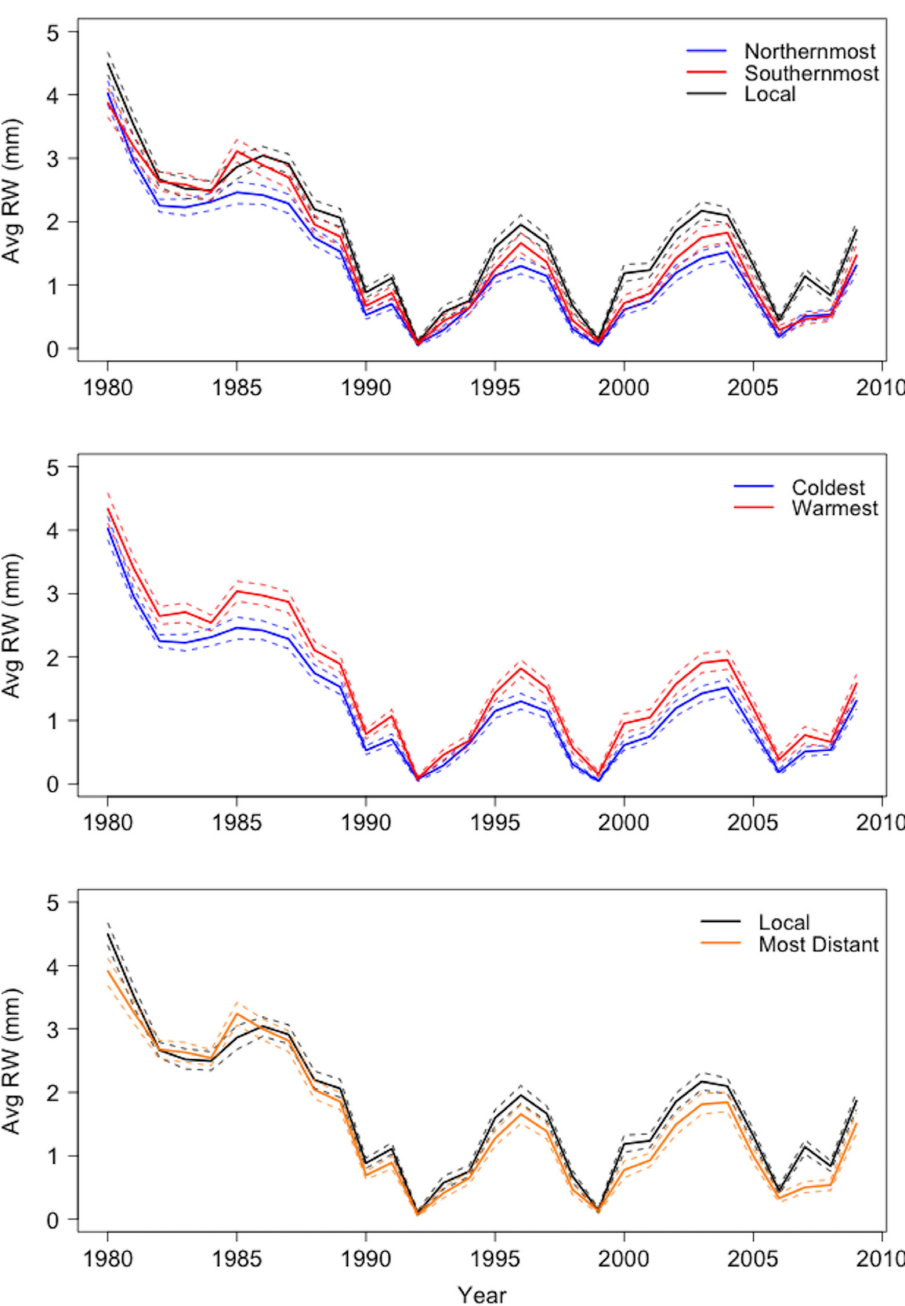
Seed source average growth through time. Average yearly tree-ring width and 95% bootstrap confidence intervals of trees from the five northernmost, southernmost, local, coldest, warmest, and most distant seed sources.

A bootstrap analysis of average ring width for individual years indicates the temporal characteristics of growth differences among seed source groups from 1980–2009 (Fig 5; S1 Table). Local seed sources had significantly wider rings relative to northernmost and southernmost seed sources in 97% and 73% of the analyzed years, respectively. Average radial growth was higher in the southern group compared to the northern group during 67% of the 1980–2009 period (Fig 5). The warmest seed sources had significantly wider average ring widths than the coldest seed sources during 93% of the same period. Local seed sources did not have significantly different average ring widths relative to the most distant seed sources in the earlier part of the record. However, local seed sources had wider average rings in 67% of the years overall; this was especially true following major missing ring events. Distant and southernmost seed source growth significantly exceeded local growth only in 1985.

### Interannual growth variability and climate response

**Fig 6:**
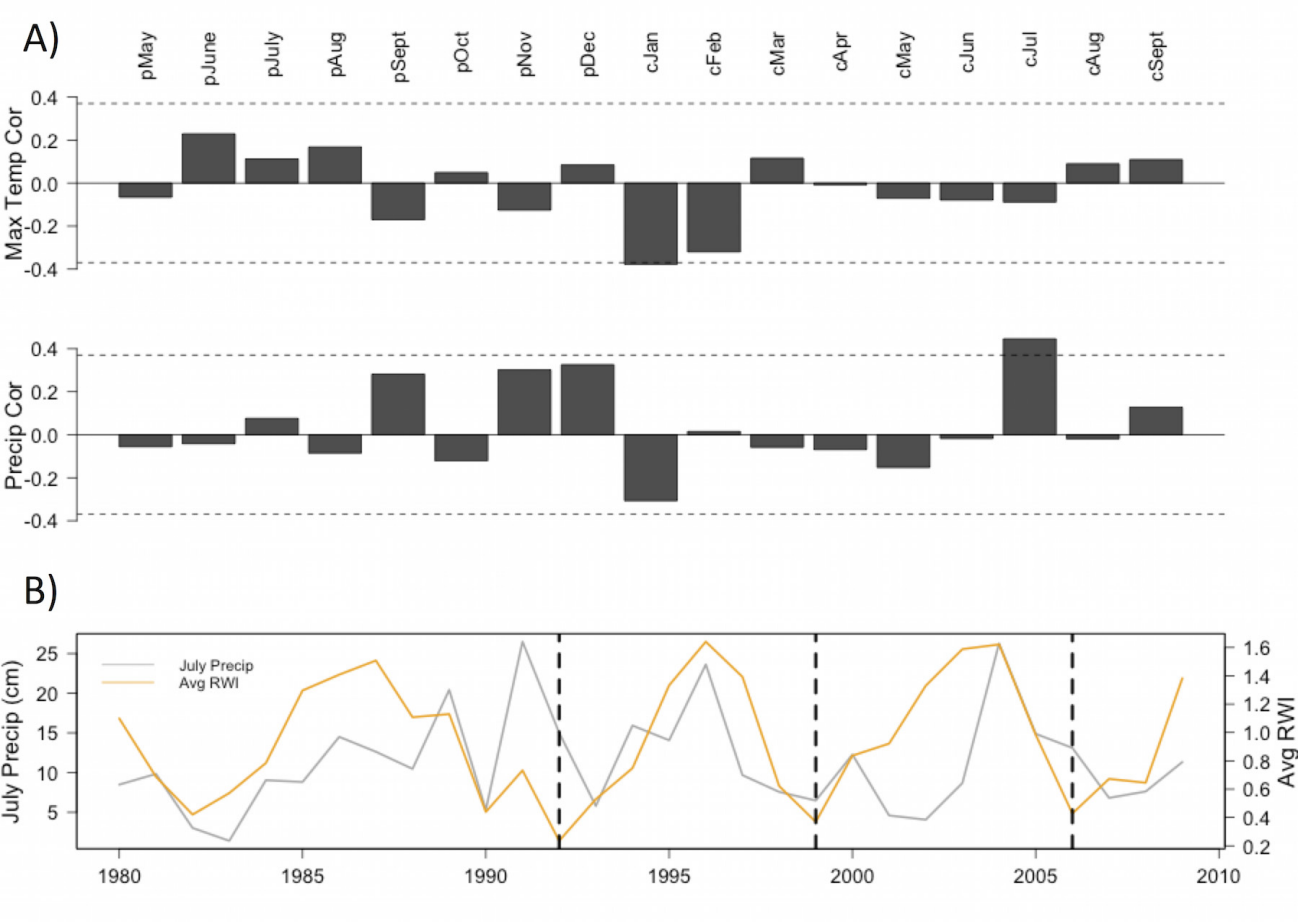
Climate response. (A) Correlations between monthly maximum temperature/monthly precipitation and average ring-width index from all seed sources combined. Pearson correlation coefficients were computed from 1980–2009 and the years 1992–1994 were not included. The dashed lines denote the 95% confidence limit (2-tailed test). (B) Comparison between July precipitation (gray) and average ring width (orange) index from 1980–2009, with dashed lines indicating major missing ring events.

A high interseries correlation within each seed source (range: 0.615–0.757) indicates that all series crossdated well (Table 1). Additionally, high Expressed Population Signal (EPS; [20]) statistics indicate a strong common signal in individual seed source chronologies (range: 0.985–0.992; Table 1). Annual growth was highly coherent among all seed source chronologies, ranging from r = 0.81–0.99 (*p* < 0.05) from 1980–2009 (S2 Fig). Further, all chronologies loaded strongly and positively onto the first and only significant principal component, which accounted for 95% of the total variance. Considering the strong similarity in interannual variability among all seed sources, we computed correlations between an average of all seed source chronologies and monthly climate data. The overall climate sensitivity was weak and the only significant monthly correlations were between the average tree-ring index and current January maximum temperatures (r = -0.38, *p* < 0.05) and current July precipitation (r = 0.45, *p* < 0.05) (Fig 6).

## Discussion

Our results demonstrate that pitch pine seed sources growing in a common plantation site have different radial growth rates, but similar interannual growth variability. Due to this common variability, we were not able to identify differences in climate sensitivity among seed sources. We found copious missing (or locally absent) rings indicating that external drivers could effectively reduce radial growth on lower tree stems of all seed sources. However, seed sources from the local, southernmost, and warmest locations had fewer missing rings and higher radial growth than seed sources from the northernmost and coldest regions.

### Missing rings, drought, and defoliation

One of the most striking and unexpected results was the high frequency of missing rings in all provenance populations. Missing rings occurred in more than 10 of the 20 years prior to our sampling, but there were particularly high rates of missing rings across all seed sources during the years 1992, 1999, and 2006 (Fig 2). The local seed sources had a lower percentage of missing rings relative to all other seed sources (Fig 3), though these populations were still strongly affected by the 1992 missing ring event. The ubiquity of missing rings found in this study is a testament to the necessity of cross-dating in tree-ring studies. Although the pitch pine trees were growing in an open plantation, stand-wide environmental disturbances clearly impacted the majority of trees in this location.

Due to the unique severity of missing rings in our series, we employed a stem analysis, as recommended by Wilmking et al. [21]. Stem analysis of the Volunteer population shows that the frequency of missing rings is highest near the base of the tree, which corresponds to the height at which core samples were taken. Partial or missing rings in the lower stem are often reported as a result of stressful environmental conditions [22–24]. Wilmking et al. [21] discuss primary physiological reasons behind missing rings in low stem sections, including reduced rates of auxin transport, or a general lack of resources that restricts cytogenesis farther away from the source of carbohydrates (needles). Considering the detected climate sensitivity to July precipitation (Fig 6A), July of 1999 had a relatively low level of precipitation (2.5 cm; Fig 6B). Therefore, it is possible that some of the missing rings may be explained by growing season drought. In contrast, the year 1992 had the highest frequency of missing rings and average moisture conditions (July precipitation = 15.01 cm). The year with the third highest number of missing rings, 2006, was also a year of slightly above-average moisture conditions (July precipitation = 13.1 cm). This suggests that other factors besides climate are likely contributing to severe growth reductions in the plantation trees.

Some years with a relatively high proportion of missing rings correspond to periods of insect defoliations in the New Jersey Pine Barrens. A defoliation signature in tree rings can be detected in multiple ways: production of white growth rings [25], a severe reduction of growth immediately following a defoliation event [26], or a failure of a tree to produce a complete ring along the entire length of the stem. Alfaro and MacDonald [26] found that Western false hemlock looper (*Nepytia freemanii*) caused growth reductions in Douglas-Fir (*Pseudotsuga menziesii*) for 1–5 years following a defoliation event. Abrupt growth reductions were also evident in this study. After radial growth suddenly declined (or ceased), recovery was slow and synchronous across many trees (e.g. 1992–1994 for all seed sources (S2 Fig)). Trees of local seed sources generally recovered a faster growth rate after missing ring events, particularly after 1999 and 2006 (Fig 5). However, after the 1992 event, local seed sources had marginally higher growth in 1993, and did not have significantly different growth in 1994 (S1 Table). This suggests that local seed sources might not have been as resilient after the 1992 missing ring event.

**Fig 7:**
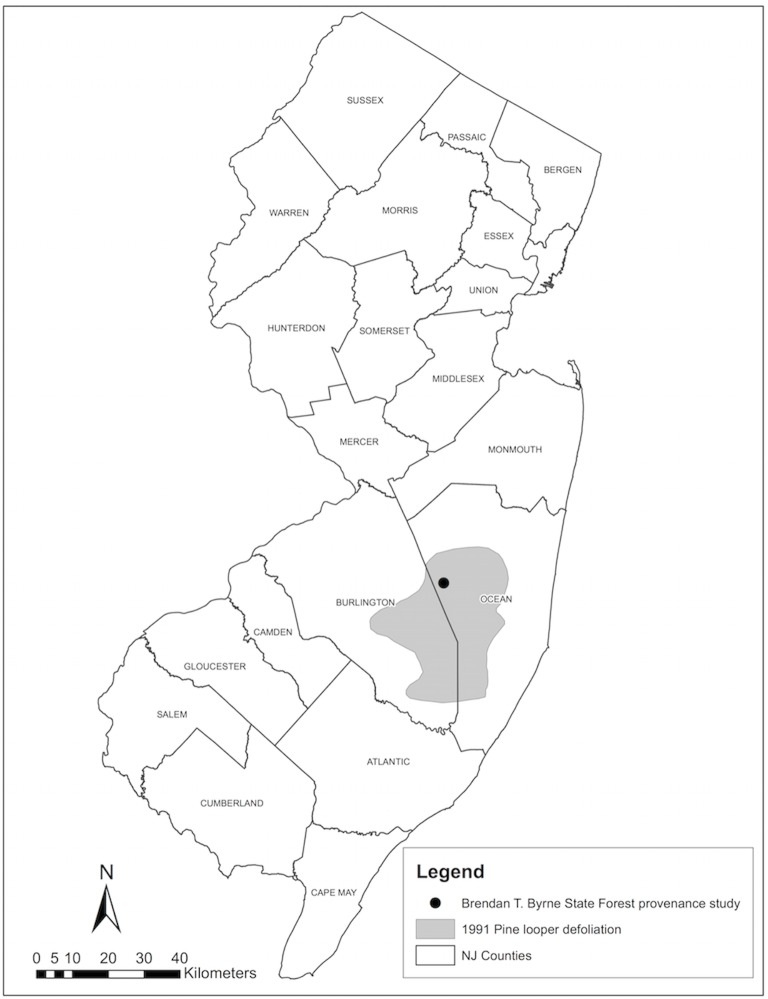
New Jersey defoliation damage in October-November 1991. The gray represents the extent of pine looper defoliation damage. The black dot is the location of the Brendan T. Byrne State Forest.

Three insect defoliators could have left a fingerprint on the forests of southern New Jersey during the period of our study: the Eastern pine looper (*Lambdina pellucidaria* Grote and Robinson), pine needleminer (*Exoteleia pinifoliella* Chambers), and gypsy moth (*Lymantria dispar* Linnaeus) [27–29]. Although there is no eyewitness account of insect defoliation in the plantation, there are numerous reports of defoliations that occurred in the region. The Eastern pine looper damaged roughly 550,000 acres of forest in New Jersey in 1991 and 1992 alone [27]. In the New Jersey pinelands near the study plantation, pine looper eggs hatched in late June of 1991 and the larvae fed from early July to early November (D. Twardus, pers. comm.). A USDA Forest Service map of pine looper damage indicates that the area around and including Brendan T. Byrne State Forest was heavily defoliated by November of 1991 (Fig 7). Autumn frosts often kill feeding pine loopers, however, the first autumn frost in 1991 arrived late and likely allowed feeding to extend longer than usual. Additionally, it was observed that the late frost allowed many of the loopers to pupate, and therefore survive and continue the cycle in the following year (D. Twardus, pers. comm.). Our tree-ring data support evidence of Eastern pine looper defoliation as there was narrow growth in 1991 when the insects emerged, followed by a high percentage of missing rings in 1992, and a slow recovery of growth after 1992 (Fig 2; Fig 5).

Similar observations were made in summer 1998. The Eastern pine looper and pine needleminer damaged 390,000 acres of pitch pine across New Jersey [30]. High-resolution aerial survey maps from 1998, provided by the USDA Forest Service, indicate that there was damage caused by the pine needleminer and pine looper within and surrounding the Brendan T. Byrne State Forest. The mature needleminer caterpillar is often present in early spring [31]. New eggs hatch in late July, and larvae continue to mine needles until they pupate in late May to early June of the following year [32]. The life cycle of the needleminer caterpillar would most likely add an additional 3–4 months of potential tree damage during a simultaneous outbreak with the Eastern pine looper. This defoliation event, in addition to drought conditions, could have contributed to the missing rings in the late 1990s across the plantation site. Reduced growth and missing rings in 2000 suggest that these trees recovered slowly.

Gypsy moth defoliation in both oak and pine-dominated stands in the New Jersey Pine Barrens has been documented [33]. Gypsy moth populations increased significantly in this region during the early 2000s and extensive defoliations occurred in late spring and early summer of 2006 and 2007 [28,29]. Though gypsy moths prefer oak species as a host species, pitch pine is considered an intermediate host [34]. Extensive gypsy moth defoliation can result in reduced annual increment of pitch pine [35], especially where pine is mixed with many oak trees or other preferred species [36]. Oak trees were occasionally removed from the study site to reduce competition, which makes it less likely that the gypsy moth was an important defoliator in the plantation. However, aerial survey maps from 2006–2009 show gypsy moth defoliation within a 5 km radius of the plantation. While the 2008 damage assessment map suggests the plantation was directly affected by these defoliators, there are no reports of gypsy moth defoliation in early June of that year within the plantation complex (T. Ledig, pers. obs.). Of the three missing ring events since 1990, the 2006–2008 event was the least severe. With pitch pine only being an intermediate host of gypsy moth, no insect outbreaks in the plantation site observed or known, and average to above-average moisture conditions, the trigger(s) of this missing ring event in the provenance plantation trees remains unknown.

### Annual growth increment and variability

Our results suggest that local seed sources performed better in terms of radial growth, which supports previous research from this provenance plantation. Kuser and Ledig [11] analyzed mean bole volume of 12-year old pitch pines from 17 seed sources, originating from New Jersey to Massachusetts. They found a negative relationship between mean volume and distance from provenance (R^2^ = 0.83, *p* < 0.05), where trees from nearby seed sources (5.3–24.8 km away) had the largest mean bole volume. Our results indicate a similar degree of local environmental adaptation (Fig 5). Indeed, local seed sources are often used for reforestation efforts (i.e. the ‘null transfer’) because in many cases, they are found to have better fitness relative to more distant seed sources under current climatic conditions [37]. Additionally, asymmetric gene flow from the core of a species range to the periphery might impede adaptation on the margins of a species range, therefore, tree populations in the center of the range might be best adapted to their local climate [3,37,38].

In common plantation sites, seed sources from particular regions sometimes outperform those from other regions. Besides local seed sources, our results indicate that the southernmost and warmest seed sources had higher average radial growth than the northernmost and coldest seed sources in the plantation during the majority of analyzed years (Fig 5). Trees from the warmest seed sources also had a significantly lower percentage of rings compared to the coldest seed sources. It should be noted that one of the five warmest seed sources in our study, Batsto (BA), is also included in the local group of seed sources, which was found to have particularly high growth rates. Even with Batsto not included in the “warmest” seed source group, this group still had significantly higher average ring widths than the coldest seed sources during 76% of the analyzed years (results not shown). Other provenance studies investigating radial increment [10] and tree height [7] have similarly shown that seed sources from warmer locations can grow better than those from colder locations in a common plantation. In some species, this is thought to reflect the negative relationship between growth rates and tolerance to freezing temperatures [5]; trees from warmer provenances might have naturally higher growth rates but lower cold tolerance than trees from cooler provenances. Such differences might allow populations from milder climates to outcompete northern populations in common garden settings. There was no discernible relationship between pitch pine growth in the plantation and provenance precipitation in our study.

PCA results and correlations among all chronologies indicated that the seed sources did not differ considerably in terms of interannual variability in radial growth. Prevalent missing rings might have contributed to the synchronization of radial growth patterns and common variability among all seed sources (S2 Fig). Tree growth across the plantation was significantly correlated to July precipitation of the growing season, a pattern previously reported for pitch pine [39]. Additionally, there was a negative correlation between average index and January maximum temperatures, though this correlation was marginally significant. This latter result is surprising considering a Pederson et al. [40] study that found positive correlations between winter temperatures and pitch pine chronologies. However, the positive winter temperature response was apparent only in sites from the northern Hudson Valley, and it was nonexistent at a site in the mid-Hudson Valley region. Therefore, the pitch pines in central New Jersey are not expected to have a strong positive winter signal given evidence that the strength of a winter temperature signal declines as one moves south from a northern range margin [8,41].

It is possible that no disparities in annual growth variability or climate response exist among the seed sources. Reports of negligible differences in seed-source sensitivities to climate are not unprecedented [8]. However, in the Cook et al. [8] study there was sometimes considerable variability between trees within each seed source, whereas we found very high series intercorrelations and a strong common signal among seed sources. Potential stand-wide disturbances, such as defoliation event(s), hypothetically synchronized growth across the plantation during certain periods. This could have hindered identification of differences in climate sensitivity among seed sources, if such differences exist.

### Implications

An important implication of this study lies at the intersection of climate, defoliators, and growth of different pitch pine seed sources planted in the Brendan T. Byrne State Forest. Based on higher growth rates and an overall lower percentages of missing rings in local seed sources, our results suggest that these seed sources might better withstand the combination of stressful environmental conditions (biotic or abiotic) in the study area. Growing pitch pine trees from non-local locations could further reduce carbon uptake relative to natively sourced trees as a consequence of defoliation events and other drivers of missing rings. However, based on our missing rings analysis and regional forest health reports, we found that all trees were strongly affected by the 1992 missing ring event and presumed pine looper defoliation. Defoliations can have significant impacts on regional carbon uptake; Clark et al. [33] quantified the influence of “transient” disturbance (i.e. defoliations) on forest carbon in oak-, mixed-, and pine-dominated stands in the New Jersey Pine Barrens and found that gypsy moth defoliations in 2006 and 2007 reduced CO_2_ uptake relative to 2005, a year with minimal defoliation.

The relationship between defoliating insects, low precipitation, and tree growth should be considered in the context of a warming climate. While temperature effects on defoliators might be negligible or species dependent [42,43], climate change is predicted to influence the phenology and spatial characteristics of insect and host-plant interactions [43]. Variability in moisture can also influence insect populations, and moisture deficits can predispose trees to insect attacks [44,45]. Warming temperatures could enhance potential evapotranspiration and trigger drought-like conditions regardless of precipitation trends [46,47]. Heat-enhanced drought might make the interaction between climate change, insect outbreaks, and tree growth more complex.

## Conclusion

The results of our study reveal some of the many factors that influence the growth of plantation pitch pine originating from different seed sources. These seed sources show differences in the rate of absolute radial growth, but do not vary significantly in annual variability. All trees, regardless of seed source, were stressed during the same years such that basal radial growth nearly ceased and slowly recovered over the course of a few years. It is hypothesized that these years of low growth are most likely the result of severe insect defoliation, low moisture availability, or a combination of both. Importantly, the high frequency of missing rings across most samples emphasizes the necessity of cross-dating for reliable tree-ring analysis. Local seed sources performed better under the extreme environmental conditions of the past three decades, as they had statistically higher growth rates during most years and fewer missing rings. The northernmost and coldest seed sources had the lowest radial growth. Tree growth response to stressful environmental conditions could have important implications for carbon dynamics in a reforested system.

## Supporting Information

S1 Fig Average ring width of all trees across all seed sources. The average ring width from 1980–2009 is shown for each tree (gray line) across all seed sources arranged based on seed source locational or climatological characteristics. The red dot indicates the mean tree-ring width value for all trees from a single seed source. The black lines are trees from the Volunteer population.
(TIFF)

S2 Fig Seed source chronologies. All seed source chronologies are arranged from north to south going down each column from left to right.
(EPS)

S1 Table Annual bootstrap growth comparison between seed source groups. X indicates that the first seed source group listed has significantly higher average growth than the second group listed (*p* < 0.05). The bottom row shows the overall percentage of significantly different years from 1980–2009.
(DOCX)

## References

[1] Lantz CW, Kraus JF. A guide to southern pine seed sources. Gen Tech Rep—Southeast For Exp Serv USDA For Serv. 1987;(SE-43):iv + 34 pp.

[2] Schmidtling RC, Froelich RC. Thirty-seven year performance of loblolly pine seed sources in eastern Maryland. For Sci. 1993;39(4):706–21.

[3] Savolainen O, Pyhäjärvi T, Knürr T. Gene flow and local adaptation in trees. Annu Rev Ecol Evol Syst. 2007;38(1):595–619.

[4] Carter K. Provenance tests as indicators of growth response to climate change in 10 north temperature tree species. Can J For Res. 1996;26(6):1089–95.

[5] Rehfeldt GE, Ying CC, Spittlehouse DL, Hamilton DA. Genetic responses to climate in Pinus contorta: niche breadth, climate change, and reforestation. Ecol Monogr. 1999 8 1;69(3):375–407.

[6] Ledig TF, Kitzmiller JH. Genetic strategies for reforestation in the face of global climate change. For Ecol Manag. 1992 7 15;50(1–2):153–69.

[7] Leites LP, Robinson AP, Rehfeldt GE, Marshall JD, Crookston NL.Height-growth response to climatic changes differs among populations of Douglas-fir: a novel analysis of historic data. Ecological Applications. 2012; 22(1):154–65. 22471081 10.1890/11-0150.1

[8] Cook ER, Nance WL, Krusic PJ, Grissom J. Modeling the differential sensitivity of loblolly pine to climatic change using tree rings In: The Productivity and Sustainability of Southern Forest Ecosystems in a Changing Environment [Internet]. Springer; 1998 [cited 2014 Jan 7]. p. 717–39. Available: http://link.springer.com/chapter/10.1007/978-1-4612-2178-4_39

[9] McLane SC, Daniels LD, Aitken SN. Climate impacts on lodgepole pine (Pinus contorta) radial growth in a provenance experiment. For Ecol Manag. 2011 7 15;262(2):115–23.

[10] Savva Y, Denneler B, Koubaa A, Tremblay F, Bergeron Y, Tjoelker MG. Seed transfer and climate change effects on radial growth of jack pine populations in a common garden in Petawawa, Ontario, Canada. For Ecol Manag. 2007 4 30;242(2–3):636–47.

[11] Kuser JE, Ledig FT. Provenance and progeny variation in pitch pine from the Atlantic Coastal Plain. For Sci. 1987;33(2):558–64.

[12] Ledig FT, Hom J, Smouse PE. The evolution of the New Jersey pine plains. Am J Bot. 2013;100:778–91. 10.3732/ajb.1200581 23515907

[13] Stokes MA, Smiley TL. An Introduction to Tree-Ring Dating. Chicago, USA: The University of Chicago Press; 1968.

[14] Regent Instruments Canada Inc. WINDENDRO for Tree-ring Analysis. 2009.

[15] Maxwell RS, Wixom JA, Hessl AE. A comparison of two techniques for measuring and crossdating tree rings. Dendrochronologia. 2011;29(4):237–43.

[16] Holmes RL. Computer-assisted quality control in tree-ring dating and measurement. Tree-Ring Bull. 1983;43(1):69–78.

[17] PRISM Climate Group [Internet]. Oregon State University; 2004. Available: http://prism.oregonstate.edu

[18] Cook ER, Krusic PJ. ARSTAN [Internet]. 2014. Available: www.ldeo.columbia.edu/tree-ring-laboratory/resources/software

[19] Cook ER, Peters K. Calculating unbiased tree-ring indices for the study of climatic and environmental change. The Holocene. 1997 1 1;7(3):361–70.

[20] Wigley TML, Briffa KR, Jones PD. On the average value of correlated time series, with applications in dendroclimatology and hydrometeorology. J Clim Appl Meteorol. 1984 2 1;23(2):201–13.

[21] Wilmking M, Hallinger M, Van Bogaert R, Kyncl T, Babst F, Hahne W, et al Continuously missing outer rings in woody plants at their distributional margins. Dendrochronologia. 2012;30(3):213–22.

[22] Harper AG. Defoliation: its effects upon the growth and structure of the wood of Larix. Ann Bot. 1913;(4):621–42.

[23] Kolishchuk V. Dendroclimatological study of prostrate woody plants. Methods Dendrochronology Appl Environ Sci Lond Kluwer. 1990;51–5.

[24] Novak K, de Luis M, Čufar K, Raventós J. Frequency and variability of missing tree rings along the stems of Pinus halepensis and Pinus pinea from a semiarid site in SE Spain. J Arid Environ. 2011 5;75(5):494–8.

[25] Hogg EH, Hart M, Lieffers VJ. White tree rings formed in trembling aspen saplings following experimental defoliation. Can J For Res. 2002 11 1;32(11):1929–34.

[26] Alfaro RI, MacDonald RN. Effects of defoliation by the Western False Hemlock Looper on douglas-fir tree ting chronologies. 1988.

[27] Robichaud B, Anderson K. Plant communities of New Jersey: a study in landscape diversity. Rutgers University Press; 1994.

[28] USDA Forest Service. New Jersey Forest Health Highlights [Internet]. 2006. Available: http://www.fs.fed.us/foresthealth/fhm/fhh/fhh_06/nj/nj_06.pdf

[29] USDA Forest Service. New Jersey Forest Health Highlights [Internet]. 2009. Available: http://www.fs.fed.us/foresthealth/fhm/fhh/fhh_09/nj_fhh_09.pdf

[30] USDA Forest Service. New Jersey Forest Health Highlights [Internet]. 1999. Available: http://fhm.fs.fed.us/fhh/fhh-98/nj/nj99.pdf

[31] Maier CT. Caterpillars on the foliage of conifers in the Northeastern United States. USDA Forest Service, Forest Health Technology Enterprise Team; 2004.

[32] Finnegan RJ. The Pine Needle Miner, Exoteleia pinifoliella (Chamb.) (Lepidoptera: Gelechiidae), in Quebec. Can Entomol. 1965 7;97(07):744–50.

[33] Clark KL, Skowronski N, Hom J. Invasive insects impact forest carbon dynamics. Glob Change Biol. 2010;16(1):88–101.

[34] Rossiter M. Use of a secondary host by non-outbreak populations of the gypsy moth. Ecology. 1987 8 1;68(4):857–68.

[35] Muzika RM, Liebhold AM. Changes in radial increment of host and nonhost tree species with gypsy moth defoliation. Can J For Res. 1999 9 15;29(9):1365–73.

[36] Schweitzer D. Gypsy Moth (Lymantria dispar): Impacts and options for biodiversity-oriented land managers. 59 pp. Nature Serve Explorer. Arlington, Virginia. 2004.

[37] Aitken SN, Yeaman S, Holliday JA, Wang T, Curtis-McLane S. Adaptation, migration or extirpation: climate change outcomes for tree populations. Evol Appl. 2008;1(1):95–111. 10.1111/j.1752-4571.2007.00013.x 25567494 PMC3352395

[38] Garcia-Ramos G, Kirkpatrick M. Genetic models of adaptation and gene flow in peripheral Populations. Evolution. 1997 2;51(1):21. 28568782 10.1111/j.1558-5646.1997.tb02384.x

[39] Cook ER, Jacoby GC. Tree-ring-drought relationships in the Hudson Valley, New York. Science. 1977 10 28;198(4315):399–401. 17809441 10.1126/science.198.4315.399

[40] Pederson N, Cook ER, Jacoby GC, Peteet DM, Griffin KL. The influence of winter temperatures on the annual radial growth of six northern range margin tree species. Dendrochronologia. 2004 12 7;22(1):7–29.

[41] Bhuta AAR, Kennedy LM, Pederson N. Climate-radial growth relationships of northern latitudinal range margin Longleaf Pine (Pinus palustris P. Mill.) in the Atlantic Coastal Plain of Southeastern Virginia. Tree-Ring Res. 2009 7 1;65(2):105–15.

[42] Williams RS, Norby RJ, Lincoln DE. Effects of elevated CO2 and temperature-grown red and sugar maple on gypsy moth performance. Glob Change Biol. 2000;6(6):685–95.

[43] Harrington R, Woiwod I, Sparks T. Climate change and trophic interactions. Trends Ecol Evol. 1999 4 1;14(4):146–50. 10322520 10.1016/s0169-5347(99)01604-3

[44] Mattson WJ, Haack RA. The role of drought in outbreaks of plant-eating insects. BioScience. 1987 2 1;37(2):110–8.

[45] Gaylord ML, Kolb TE, Pockman WT, Plaut JA, Yepez EA, Macalady AK, et al Drought predisposes piñon–juniper woodlands to insect attacks and mortality. New Phytol. 2013;198(2):567–78. 10.1111/nph.12174 23421561

[46] Cook BI, Smerdon JE, Seager R, Coats S. Global warming and 21st century drying. Clim Dyn. 2014 3 6;1–21.

[47] Cook BI, Ault TR, Smerdon JE. Unprecedented 21st century drought risk in the American Southwest and Central Plains. Sci Adv. 2015;1(1):e1400082 10.1126/sciadv.1400082 26601131 PMC4644081

[48] Little Jr EL. Atlas of United States trees. Volume 1. Conifers and important hardwoods. Miscellaneous publication 1146. US Dep Agric For Serv Wash DC. 1971.

