# Supplementary figures and images for: Missing Rings, Synchronous Growth, and Ecological Disturbance in a 36-Year Pitch Pine (Pinus rigida) Provenance Study
Source: PLoS One. 2016 May 16;11(5):e0154730. doi: 10.1371/journal.pone.0154730 (PMC4868262; doi:10.1371/journal.pone.0154730)

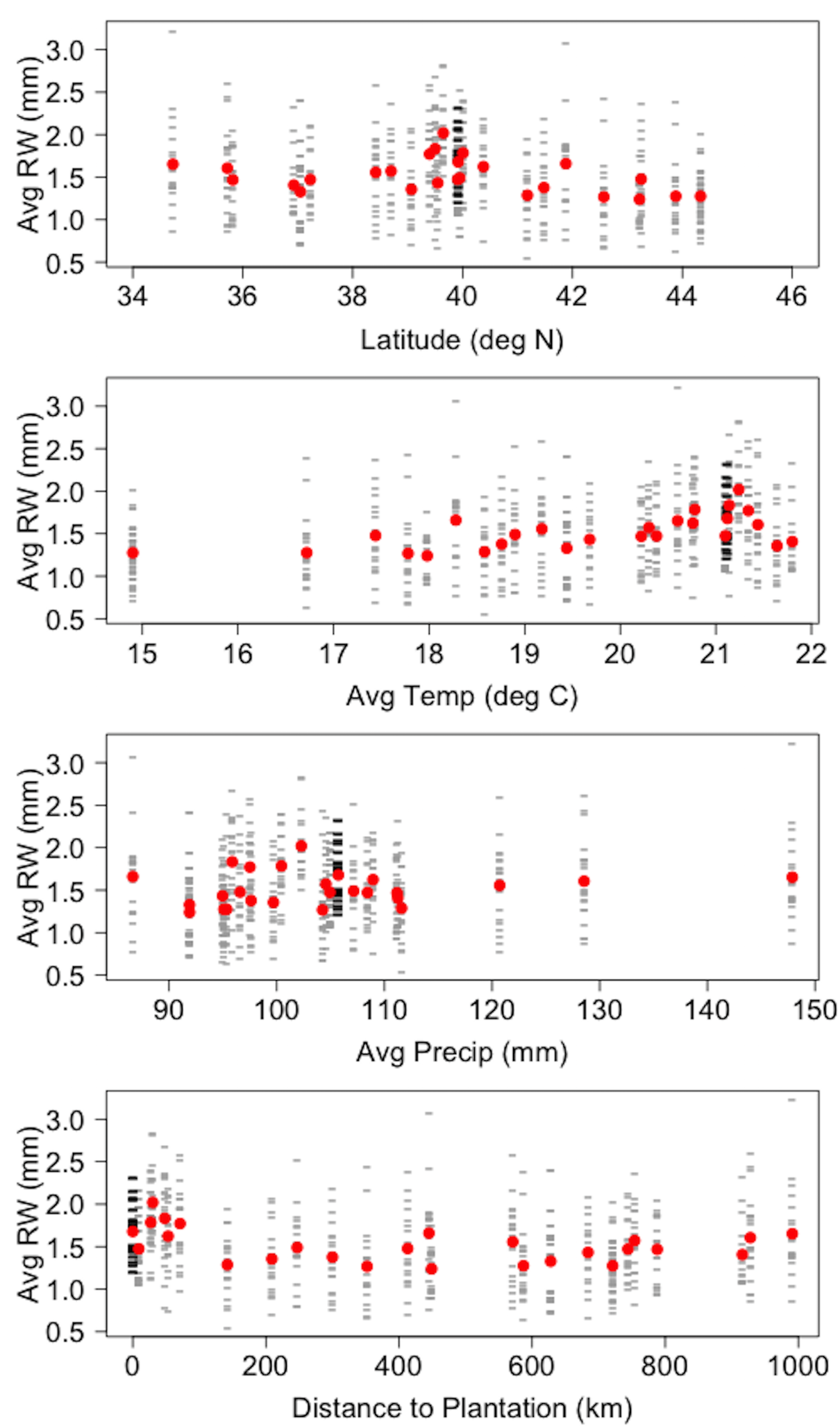

Supplement: S1 Fig — The average ring width from 1980–2009 is shown for each tree (gray line) across all seed sources arranged based on seed source locational or climatological characteristics. The red dot indicates the mean tree-ring width value for all trees from a single seed source. The black lines are trees from the Volunteer population. (TIFF) [file pone.0154730.s001.tiff]
