# Supplementary material for: Missing Rings, Synchronous Growth, and Ecological Disturbance in a 36-Year Pitch Pine (Pinus rigida) Provenance Study
Source: PLoS One. 2016 May 16;11(5):e0154730. doi: 10.1371/journal.pone.0154730 (PMC4868262; doi:10.1371/journal.pone.0154730)
Supplement: S1 Table — X indicates that the first seed source group listed has significantly higher average growth than the second group listed (p < 0.05). The bottom row shows the overall percentage of significantly different years from 1980–2009. (DOCX) [file pone.0154730.s003.docx]

| **Year** | **Local - North** | **Local - South** | **South - North** | **Warm - Cold** | **Local - Distant** |
| --- | --- | --- | --- | --- | --- |
| 1980 | X | X |  | X | X |
| 1981 | X | X | X | X | X |
| 1982 | X |  | X | X |  |
| 1983 | X |  | X | X |  |
| 1984 | X |  | X | X |  |
| 1985 | X |  | X | X |  |
| 1986 | X |  | X | X |  |
| 1987 | X | X | X | X |  |
| 1988 | X | X | X | X |  |
| 1989 | X | X | X | X | X |
| 1990 | X | X | X | X | X |
| 1991 | X | X | X | X | X |
| 1992 |  |  |  |  |  |
| 1993 | X | X | X | X | X |
| 1994 | X |  |  |  |  |
| 1995 | X | X |  | X | X |
| 1996 | X | X | X | X | X |
| 1997 | X | X | X | X | X |
| 1998 | X | X | X | X | X |
| 1999 | X |  | X | X |  |
| 2000 | X | X |  | X | X |
| 2001 | X | X |  | X | X |
| 2002 | X | X | X | X | X |
| 2003 | X | X | X | X | X |
| 2004 | X | X | X | X | X |
| 2005 | X | X |  | X | X |
| 2006 | X | X | X | X | X |
| 2007 | X | X |  | X | X |
| 2008 | X | X |  | X | X |
| 2009 | X | X |  | X | X |
| **Percent Sig** | **97%** | **73%** | **67%** | **93%** | **67%** |

**S1 Table:** **Annual bootstrap growth comparison between seed source groups.** X indicates that the first seed source group listed has significantly higher average growth than the second group listed (*p* < 0.05). The bottom row shows the overall percentage of significantly different years from 1980-2009.
